# New Tools in Orthology Analysis: A Brief Review of Promising Perspectives

**DOI:** 10.3389/fgene.2017.00165

**Published:** 2017-10-31

**Authors:** Bruno T. L. Nichio, Jeroniza Nunes Marchaukoski, Roberto Tadeu Raittz

**Affiliations:** Department of Bioinformatics, Professional and Technical Education Sector, Federal University of Paraná, Curitiba, Brazil

**Keywords:** orthology prediction, bioinformatics, comparative analysis, genomic dynamics, phylogeny

## Abstract

Nowadays defying homology relationships among sequences is essential for biological research. Within homology the analysis of orthologs sequences is of great importance for computational biology, annotation of genomes and for phylogenetic inference. Since 2007, with the increase in the number of new sequences being deposited in large biological databases, researchers have begun to analyse computerized methodologies and tools aimed at selecting the most promising ones in the prediction of orthologous groups. Literature in this field of research describes the problems that the majority of available tools show, such as those encountered in accuracy, time required for analysis (especially in light of the increasing volume of data being submitted, which require faster techniques) and the automatization of the process without requiring manual intervention. Conducting our search through BMC, Google Scholar, NCBI PubMed, and Expasy, we examined more than 600 articles pursuing the most recent techniques and tools developed to solve most the problems still existing in orthology detection. We listed the main computational tools created and developed between 2011 and 2017, taking into consideration the differences in the type of orthology analysis, outlining the main features of each tool and pointing to the problems that each one tries to address. We also observed that several tools still use as their main algorithm the BLAST “all-against-all” methodology, which entails some limitations, such as limited number of queries, computational cost, and high processing time to complete the analysis. However, new promising tools are being developed, like OrthoVenn (which uses the Venn diagram to show the relationship of ortholog groups generated by its algorithm); or proteinOrtho (which improves the accuracy of ortholog groups); or ReMark (tackling the integration of the pipeline to turn the entry process automatic); or OrthAgogue (using algorithms developed to minimize processing time); and proteinOrtho (developed for dealing with large amounts of biological data). We made a comparison among the main features of four tool and tested them using four for prokaryotic genomas. We hope that our review can be useful for researchers and will help them in selecting the most appropriate tool for their work in the field of orthology.

## Introduction

Finding the homology relationship between sequences is an essential step for biological research. Within homology, the orthology analyses, that consist in finding out if a pair of homologous genes are orthologs—i.e., resulting from a speciation—or paralogs—i.e., resulting from a gene duplication—is very important in computational biology, genome annotation, and phylogenetic inference (Ullah et al., 2015). Because of this, the highlight of the present research was the development of computational tools that aim at facilitating this field of study.

The process of orthologs detection, besides being closely related to comparative analysis and genomic dynamism, is also an extremely important field of study for helping to improve the functional annotation of various organisms (Kim et al., 2011) and it is still very important to elucidate processes evolving the appearance of species (Wang et al., 2015). An accurate orthology recognition is an essential step for comparative genomic researches (Petersen et al., 2017) and then, in some cases, there is a need for tools that analyze closely related species by pangenomas (Fouts et al., 2012) or for the creation of tools that use different strategies like the post-translational modifications proteins (PTMs) for a better orthology inference (Chaudhuri et al., 2015).

Since the early studies involving the establishment of techniques for inferring orthology, the main difficulty was the lack of a methodology and of a tool to be fully reliable in assemblying orthologs sets of data. It was only in 2007, that the first study about the sensitivity, accuracy, and performance methods in detecting these groups arose (Altenhoff and Dessimoz, 2009) thus consecrating methodological “gold standards” as adaptations of—among others the Markov Cluster Algorithm models of the Basic Alignment Search Tool (BLAST) algorithm (Chen et al., 2007); of Reciprocal Best Hits (RBH) (Zielezinski et al., 2017); of Correlation Coefficient-based Clustering (COCO-CL), COCO-CL (Raja et al., 2006); of Automatic Clustering of Orthologs and In-paralogs (InParanoid) (O'Brien, 2004) leading to the appearance of various tools such as the a Markov Cluster algorithm for grouping proteins into multiple species of ortholog groups (OrthoMCL) (Li et al., 2003). BLAST tool and adaptations were consolidated in subsequent studies (Kristensen et al., 2011) resulting in several publications and in the creation of large biological databases containing Ortholog *Clusters* such as the *Clusters* of Orthologs Groups/euKaryotic Orthologous Groups (COG/KOG), Ortholog Data Bank (OrthoDB), and eggNOG (Kuzniar et al., 2008).

However, it is difficult to detect orthologous groups (Tekaia et al., 2016) and there is no effective tool for detecting these groups, because the accumulation of evolutionary dynamic events tends to difficult the recognition of true orthologs among homologs (Novo et al., 2009), but rather a set of tools that meet certain computational demands and interests of its users (Altenhoff and Dessimoz, 2009). Also perceived was the need of many improvements still to be made for a more accurate orthology prediction using these tools building or upgrading the ortholog relationships between genomes requires a lot of computational effort and a lot of time (Tabari and Su, 2017), besides, relating orthology between organisms having distant kinship origins, for instance, still remains a remarkable challenge (Chen et al., 2010).

All of this is gets worse when there is a need to include a large number of sequences to be analyzed in order to infer orthology (Bitard-Feildel et al., 2015). Another problem is the application of a high-level of programming knowledge on the part of researchers to analyze data, which hinders the smoothness of the work flow. Some methodologies and tools, like the consolidated as BLAST all-vs.-all (Schreiber and Sonnhammer, 2013), RBBH (Gupta and Singh, 2015), OrthoMCL (Chen et al., 2007), demand a high computational cost (Linard, 2011) that will add weigh on the capabilities of normal hardware and will end up requiring access to the resources of supercomputers (Lechner et al., 2011). Another factor that directly influences the demand for better tools is the ever-increasing number of genomes that are deposited in large biological sequences in databanks and that can be compared simultaneously (Muir et al., 2016). This requires more efficient software tools (Curtis et al., 2013) because those such as BLAST and InParanoid fail when orthology is involved, but the level of conservation among orthologous is low, and therefore this requires a sophisticated manual intervention and makes it difficult to automate the process (Wagner et al., 2014). Besides all that also comes the need to develop tools to improve the sensitivity in detecting orthologous groups (Emms and Kelly, 2015).

Those are the most important needs and because of them several research groups are putting in great effort to develop new tools to improve and facilitate analysis involving orthology and may also contribute to advances in later studies. Therefore, the latest tools already available should gain prominence in the scientific field. Reviews of recent ortholog tools are gaining prominence, so much that came the first review tool involving homology pan genomes by Vernikos et al. (2015) and Xiao et al. (2015). A great number of programs are available for supra-genome analysis but each of them suffers from one or the other limitations leaving gaps for further improvement (Chaudhari et al., 2016).

In order to compile our review, we focused on the most recent tools that have been developed with high expectatives for the study of orthologs, in order to bring the lastest advances in the development of more effective, fast, and multi-tasking tools for the processing of homologous ortholog data sequences.

## Highlighting main tools and methodologies

**Figure 1:**
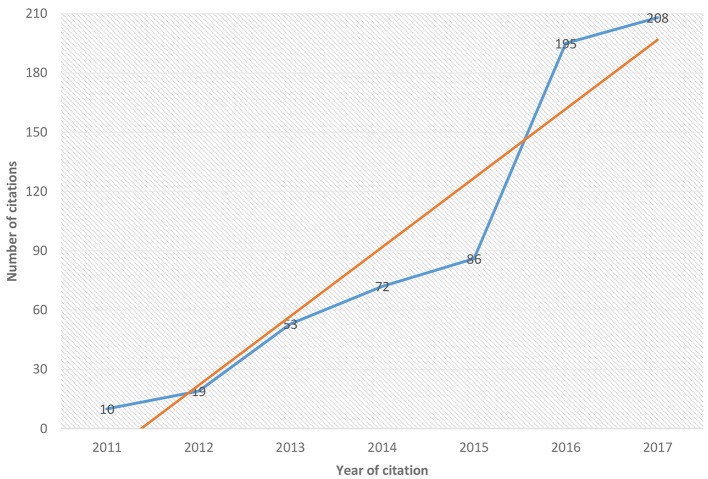
Growth of number of the citations by the new ortholog tools from 2011 to 2017. A brief relationship between the number of citations along the years. It is observed that the new softwares to use and development in the prediction of ortholog groups has been increasing in recent years since 2011 until 2017. The blue line represents the citation of emergent tools in orthology inference for each year and the red line shows the tendency to increase.

Due the importance and the growth in studies aimed at the development of new orthology techniques, since 2014 we have been monitoring various techniques of various techniques and computational tools for predicting protein or orthologous genes at the most different levels of study. After 2011 and up to the present day, we have observed a growing number of new computational tools emerging in publications as was demonstrated in Figure 1 and in Supplementary Figure 1 mainly in the last years.

The information derived from multiple banks of scientific publications (such as NCBI PubMed, Biomed Central, BMC bioinformatics, Google Scholar, and ExPASy) were filtered in a way that would allow us to find the lastest tools, softwares or recent packages that were aimed at resolving the issue of creation and consolidation of orthologous groups, but that would also solve problems left by older methodologies and tools. Each individual article was carefully examined analyzing contents of abstracts and scientific magazines, totaling more than 600 articles in order to compile the present review as demonstred in Supplementary Figure 2. Many, among the collected items, belong to BioMed Central, Oxford Journals of Bioinformatics Session, Evolutionary Bioinformatics, and Science Journals.

**Figure 2:**
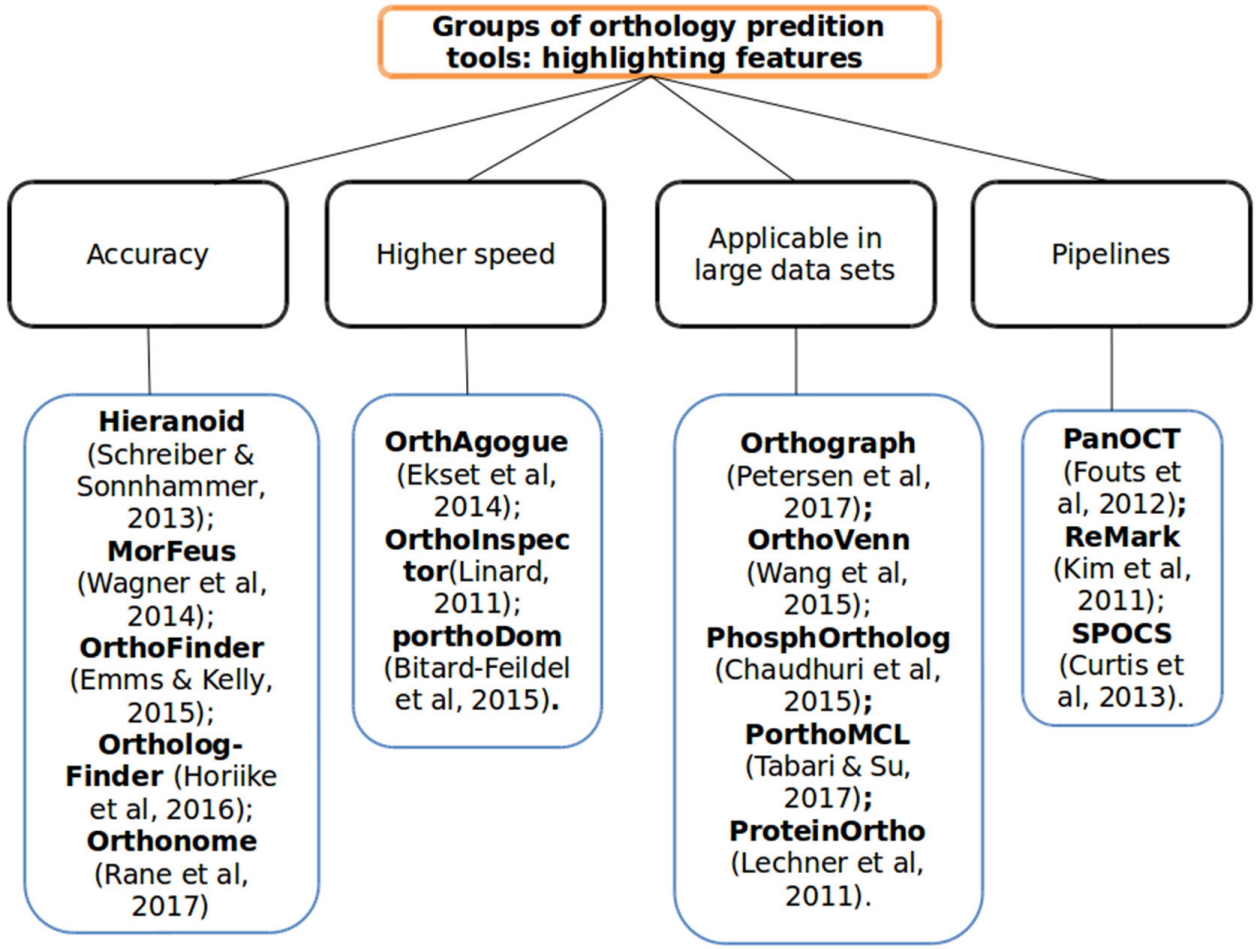
The tools overview method and its main features to orthology prediction. The ortholog tools subdivided into four categories according of each tool characteristic to show better its main applicability in orthology predictions context: in better accuracy, higher speed detection, large data set, or automation of process (pipelines).

In this study, we found several papers highlighting tools that have proved promising for incorporating new methodologies or adapting already consolidated ones. Some even include specific algorithms developed in consideration of the fact that there are various problems to be solved in ascertaining orthology between organisms—such as we characterized in Figure 2. Hereafter, we list 16 among the more recent major tools developed or in development highlighting their key features, described—in the following paragraphs and in Tables 1, 2,—algorithms involved, advantages and disadvantages and possible tool solutions in order to minimize specific problems encountered in the prediction of orthologs.

**Table 1:** Software tools features for Ortholog studies since 2011 at 2017.

**Tool**	**Main Features**	**Platform**	**Implementation**	**Disponibility**	**Year of release**
Hieranoid	Combines an efficient graph-based methodology with aspects of compute-intensive tree-based methods to infer orthology	Linux/Unix; web-server	Perl, BioPerl, BLAST, Muscle and Kalign	http://hieranoid.sbc.su.se/	2013
MorFeus	Calculates a network score to the resulting network orthologs to find remotely stored orthologs proteins	Linux/Unix; web-server:	Python, biopython, networkx, gnuplot and BLAST+	https://omictools.com/morfeus-tool	2014
OrthAgogue	High speed of the homology relationships in large data sets	Linux/Unix	BLAST, cmph and TBB	https://code.google.com/p/orthagogue/	2013
OrthoInspector	Agile detection of orthology and in-paralogy incorporing a unique algorithm	Cross-platform (Java)	BLASTP+ package, JAVA, MySQL	http://www.lbgi.fr/orthoinspector/	2011
OrthoFinder	Solves the fundamental biases in whole genome comparisons and improves inference accuracy in ortholog groups	Linux/Unix	BLASTP+ package, python, MCL graph clustering algorithm, MAFF and Fastree	http://www.stevekellylab.com/software/orthofinder	2015
Ortholog-Finder	Identifies genuine orthologs among distantly related species by phylogenetic analysis using ORF data	Linux/Unix	BLAST+, MAFFT, BioPERL, OrthoMCL, JAVA, ClustalW	http://www.grl.shizuoka.ac.jp/~thoriike/Ortholog-Finder	2016
Orthograph	Developed for a large data set maintaining the high sensitivity and accuracy with BRH approachs	Linux/Mac OS X	BLAST +, PERL, MySQL, MAFF, SWIPE	https://mptrsen.github.io/Orthograph/	2017
Orthonome	Designed to boost the accuracy of multiple-species in ortholog predictions and reduce the trade-off between ortholog captures rates	Web-Server	Linux Bash, C++, Perl and Python package	http://www.orthonome.com/	2017
OrthoVenn	Makes relationships of orthologous clusters across multiple species using Venn Diagram	Web-server	BLAST and MCL algorithms	http://aegilops.wheat.ucdavis.edu/OrthoVenn/	2015
PanOCT	Automates clusters of orthologs for pan-genomic analysis of bacterial strains and closely related species	Linux/Unix	BLAST+, PERL	https://sourceforge.net/projects/panoct/	2012
PhosphOrtholog	Developed for mapping of orthologs by protein PTMs by cross-species	Web-Server	BLOSUM62, comma separated file format (.csv) input	http://www.phosphortholog.com/	2015
PorthoDom	Developed to speed up the detection of orthologs protein using domain sequences	Cross-platform	C program, Python, pFam Database, HMMER	http://www.bornberglab.org/pages/porthoda	2015
PorthoMCL	Designed for find orthologs in a large number of genomes	Linux and Unix (OS X)	BLAST, PERL, Python, MCL	http://ehsun.me/go/porthomcl/	2017
ProteinOrtho	Dealing with hundreds of bacterial species in set containing millions of proteins using low computer memory	Linux/Unix 64 bits	BLAST+, PERL, and Python	http://www.bioinf.uni-leipzig.de/Software/proteinortho/	2011
ReMark	Identifies orthologs automatically by a parameter adjustment according to the user's interest	Cross-platform (Java)	Recursive and a Markov clustering (MCL) algorithms, Reciprocal BLAST Best Hits (RBBH), JAVA	http://dasan.sejong.ac.kr/~wikim/notice.html	2011
SPOCS	Ortholog prediction on graph method based to generate a table can provide a visualization of the relationships between the orthologs groups	Web-Server; Linux/Mac OS X	BLAST+, C++ libraries	http://cbb.pnnl.gov/portal/tools/spocs.html (web) http://cbb.pnnl.gov/portal/software/spocs.html (installer)	2013

**Table 2:** Software tools usabilities.

**Tool**	**Advantages**	**Disadvantages**
Hieranoid	Uses hierarchical approaches to ortholog inference based on InParanoid method. Its uses BLAST or not (Usearch – optionally).	Needs a lot of dependences to runs locally: Perl, BioPerl, HHSearch, BLAST or Usearch algorithms. Limit of queries on Web-Server uses.
MorFeus	Uses symmetrical best hits and orthology network scoring to detect remotely conserved orthologs. Available in web-server and locally.	A lot of dependences such BLAST (locally) to runs and it is not updated since 2014.
OrthAgogue	A multithreaded application for massive datasets with high-speed estimation of homology relations.	The input file be a tabular file generated by the BLAST algorithm is needed. BLAST package is necessary.
OrthoInspector	Incorporates an original algorithm, facilitate data query, and process automation.	Creation of a Database in Postgresql or MySQL and BLAST dependences.
OrthoFinder	Easy command that uses as input a multiFASTA file (one per species) minimizing the bias of the length, previously undetected gene in orthogroup.	Needs a lot of dependences including BLAST and MCL algorithms to run.
Ortholog-Finder	A program that identifies genuine orthologs among distant species using HGF filters for phylogenetic analysis	A lot of dependences and the program does not support the maximum likelihood or Bayes methods.
Orthonome	The algorithm provides a superior combination of ortholog capture rates and accuracy on draft or complete drosophilid genomes.	Limited number of queries (web) and depends the assemblies and annotations improves to better performance.
OrthoVenn	Visualization using the interactive Venn diagram in the generated clusters views. Brings Gene Ontology information with each protein functions.	Only web-server, limitation of queries.
Orthograph	A program easy to install that facilitates comparative analyses of transcriptomic and other coding sequence for comparative genomic analyses.	Needs a lot of dependences to run like BLAST, MySQL, MAFFT, HMMer, SWIPPE to runs.
PanOCT	Procariotic uses, Orthologs and co-orthologs relationships.	Depends on the PERL packages, BLAST+ and is limited to an analysis of up to 25 genomes.
PhosphOrtholog	Mapping between orthologous protein species from post-translational modifications (PTMs). Uses UniProt/Swiss-Prot DB reference.	Exclusively on the Web, it needs comma separated file format and it is restricted to just the proteomas of human, mouse, rat, fly.
porthoDom	Uses protein domain to speed up proteinOrtho. Uses Pfam anotation to accuracity.	Its a bit laborious to be performed, needs a lot packages, Pfam database and HMMER package, in addition to the ProteinOrtho tool.
PorthoMCL	Capability by identifying orthology in large number of genomes.	Although fast and easy tool, requires BLAST, PERL, and Python package.
ProteinOrtho	Reduces the amount of memory needed to create orthologs groups, finds co-orthologs on big banks containing different species.	Depends on libraries such BLAST +, PERL, and Python to run.
ReMark	Makes the process more automated, using adjustment according to the user's interest.	The tool is not updated since march 2011. It Needs BLAST and JAVA dependences.
SPOCS	Flexibility and automates the process of orthologs detection, without the need for multiple steps.	It needs boost C++ and BLAST previously installed and generates text or HTML outputs.

### Hieranoid: a hierarchical approach to accurate orthology inference

The growing number of large-scaled datasets is limited by the most of the tools in orthology inference and often it is necessary that accuracy is penalized. Hieranoid is a graph-based method published in 2013 that uses hierarchichal approaches to orthology inferences to keeps the performance (Schreiber and Sonnhammer, 2013) and improves the accuracy in orthology inference. Its pipeline is addressed to solutionate three main problems in orthology analysis: (1) scalability: reducing the computational complexity of similarity searches; (2) accuracy: by more fine-grained orthology inference by building hierarchical groups; and (3) Multi-species, extending the InParanoid algorithm to infer multi-species ortholog groups (Schreiber and Sonnhammer, 2013).

Developed in Perl, The Hieranoid tool is available in 1.0 and 2.0 versions and the version 1.0 has been deprecated since august 2013. The software is available on Linux to runs locally -depends some system requirements as Perl, BioPerl, Usearch (Edgar, 2010) or Blastall and HHSearch (Söding, 2005), MUSCLE (Edgar, 2004) or Kalign (Lassmann and Sonnhammer, 2005)- and disponible on web server platform. The user account with a proteomes database to assist orthology researches (only in version 2.0).

### morFeus: the use of symmetrical best hits and orthology network scoring to detect remotely conserved orthologs

The morFeus tool presents, as its key strategy, the search of remotely conserved orthologous groups. Morfeus selects sequences based on their alignment similarity of query using orthology tests based on research iterative reciprocal BLAST mode and calculates a network score to the resulting network orthologs which is a measure dependent on *e-value* implementation (Wagner et al., 2014).

Given the variability between compared sequences and heuristics of BLAST, the *e-value* aims at providing users with the assurance that the score given to a particular *hit* did not occur randomly (Korf et al., 2003). The performance of morFeus is comparable to other state-of-the-art orthology methods (Wagner et al., 2014). Besides, some of its results have already been experimentally demonstrated by developers that proved equivalent in organisms with comproved orthology thus fulfilling the criteria of the orthology-function conjecture (Wagner et al., 2014). This tool can be used both locally, and in this case only on Linux platforms, and via web -service.

### OrthAgogue: detection of orthology relations in a large data set of sequences with agility

One of the main problems concerning the most discussed in tools of orthology clustering genes and proteins analyses is the low computational performance and the high consumption of time that these tools need (Ekseth et al., 2014). Considering this context, orthAgogue was developed and published in 2013. The tool works in multithreaded and is concerned with determining at high speed relationships between sequences of genes or proteins of various species operating through a flexible and easy command line interface. The best high-scoring pairs in BLAST output (HSP) is applicated in its algorithm to search orthologous groups (Ekseth et al., 2014).

In one of the papers that was considered to copile this review, orthAgogue is compared to the OrthoMCL tool, and, among other things, it point at computational limitations of the “gold standard” tool, such as the high consumption of RAM and processing when there is need to work with large volumes of data. Therefore, orthAgogue is particularly convenient when working on large amounts of data with computers of limited capabilities. OrthAgogue is available for Linux platforms, being a tool developed in C+ language.

### Orthoinspector: comprehensive visual exploration in orthology and paralogy analysis

The OrthoInspector is a software system, which incorporates a unique algorithm for rapid detection of orthology and inparalogy between different species (Linard, 2011). First, the results of a BLAST “*all-vs. all”* is provided by the user and is parsed to find all the BLAST best hits for each protein and to build the groups of inparalogs (Linard, 2011). After that, the inparalog groups for each organism are compared in a pairwise fashion to define potential orthologs and/or in-paralogs. In the end, best hits that contradict the potential orthology between entities are detected (Linard, 2011). In comparison with traditional methods, like orthoMCL and InParanoid, the software shows improvements in the detection sensitivity with a minimal loss of specificity (Linard, 2011). Besides, the biggest difference of the package is that multiple visualization tools have been developed to facilitate analysis and study in depth based on own estimates, which allows for greater ease of consultation of the obtained data.

The OrthoInspector package, developed in Java, is compatible with any operating system, provided one has the JVM (Java Virtual Machine) preinstalled on the Operating System (OS). The tool is still in development, with a version 2.0 that has been available since 2014.

### OrthoFinder: solves fundamental biases in genomic comparisons improving accuracy in ortholog groups

OrthoFinder is an algorithm which aims to solve the bias accuracy in detecting orthologous groups. For this algorithm is divided into several stages involving BLAST “all-against-all” phylogenetic tree construction and use of MCL algorithm divided into follow step (Emms and Kelly, 2015): (1) The “unknown orthologous groups” that the algorithm must recover, shown as a gene tree; (2) the BLAST performs in all genes against all genes; (3) gene length and phylogenetic distance normalization of BLAST bit scores to give the scores to be used for ortholog group inference; and (4) selection of putative cognate gene-pairs from normalized BLAST scores (Emms and Kelly, 2015).

Using sets of real reference data demonstrated that OrthoFinder is more accurate than other methods of inference of orthologous groups already consolidated, such as OrthoMCL, TreeFam, eggNOG e OMA, between 8 and e 33%. The methodology of this tool is based on the fact that the group contain all the genes descendants from a single gene in the last common ancestor of the species whose genes are being analyzed (Emms and Kelly, 2015). This setting prevents confusing shared ancestry with other criteria that are not equivalent, such as the functional conservation (Emms and Kelly, 2015).

The tool started to be developed in 2003, was patented in 2015 (US20150284796), but only in 2015 the paper relative to the contributions was submitted. It is a simple algorithm, light and easy to use in Linux environment.

### Ortholog-Finder: a pipeline for building a ortholog data set

To seek ortholog data sets for performing phylogenetic analysis by using all openreading frame data of species, ortholog-Finder was developed (Horiike et al., 2016). Identifying genuine orthologs among distantly related species it is the main feature, focusing on five types of filtering genes to obtain through Horizontal Gene Transfer (HGT) and out-paralogs to predict orthologous groups: (1) HGT filtering; (2) Out-paralog filtering; (3) Classification of tree data; (4) Tree splitting; and (5) *E*-value changing (Horiike et al., 2016). After HGT filtering, the inferred HGT sequences and non-HGT sequences are saved separately, and the data can be used for other analyses, however, the software does not support the maximum likelihood or Bayes methods because the calculation for choosing the optimal substitution model and constructing phylogenetic requires a long time (Horiike et al., 2016).

It is downloadable to Linux/Unix platforms (it was tested on Ubuntu 12.04 LTS and CentOS 6.5) and requires BLAST+, FastTree, multiple sequence alignment program (MAFFT), conserved blocks program (Gblocks), BioPERL, EMBOSS tools, MCL, OrthoMCL, and JAVA Runtime package to runs.

### Orthograph: a versatile strategy for mapping transcription elements to orthologous groups of genes

The Orthograph makes orthology prediction using a graph-based method. The pipeline applies and searches the RBH strategy given the complete information of the organism gene when the repertoire is available (e.g., RNAseq; Petersen et al., 2017). Orthograph uses the hidden Markov model and its maps nucleotide sequences to the globally best matching cluster of orthologous genes, thus enabling researchers to conveniently and reliably delineate orthology and paralogy from transcriptomic and genomic sequence of data (Petersen et al., 2017). Orthograph solves the problems suffers from issues that may cause problems in downstream analyses and is focused in RNAseq analysis being a easy to use tool and flexible to user.

The software is written in Perl and its package runs locally an Unix/Linux systems (including OS X) but dependences are needed to run as BLAST + package, MySQL, Perl, Hidden Markov Models (HMMER), MAFFT, SWIPE.

### Orthonome: a tool for accuracy of orthology prediction on multi-species to draft or complete genomes

Orthonome was developed to increase accuracy among species in ortholog prediction and to reduce the trade-off between ortholog capture rates (denominated as recall). Published in 2017, the pipeline is divided in five main steps: (1) Processing input files: the CDS and pepitides annotations are submitted on BLASTp comparison between each combination of species; (2) Pairwise species comparisons and inparalog detection: to adaptative identification of ancestrality and inparalogs genes; (3) Smith-Waterman score adjustment: is necessary to scale SW score to account for fragment length bias in local alignment; (4) Ortholog prediction: statistics to ortholog prediction and correction. Species phylogenetic constructions and Multispecies tree reconciliation; (5) Orthogroup clustering and evolutionary classification: using a high-throughput ortholog assignment system (MSOAR) to orthogroups identification and classification (Rane et al., 2017).

Orthonome produces tabulated and databasetool-friendly formats which are utilized to build the web interface with an online database (Rane et al., 2017). The current implementation includes output data from the 20 Drosophila species analyzed in previous studies. Its pipeline was developed in Unix bash and C++ scripts assisted by toolkits in Perl and Python and available on web-server.

### OrthoVenn: a web server for comparison and annotation of ortholog genomes across multiple species

Focusing on comparative genomics study, OrthoVenn implemented in Java, tries to illustrate, using the Venn diagram to create an overlap between the clusters of orthologous groups, and the function and evolution of proteins in various species (Wang et al., 2015). OrthoVenn is a “Web-only tool” for viewing wide comparisons of orthologous groups of genomes with an interactive view of the Venn diagram and provides a summary of high-level functions for sets that overlap, or do not overlap, orthologous genes. It is a tool composed of several methods such as MCL, BLAST all vs. all and besides, for the identification of hypothetical orthology, OrthoVenn uses the OrthAgogue tool for identifying orthology and inparalogy relations (Wang et al., 2015).

OrthoVenn is available on web server and it allows personalized protein analysis from defined species on the part of the user. The software includes deep views of clusters using various analysis tools.

### PanOCT: automated clustering of orthologous groups for supra-genomic analysis of bacterial strains or in closely related species

The pan genoma analysis of prokaryotes species or strains closely related, is the main function of the Pan Genome Ortholog Clustering Tool (PanOCT). It is a specific tool to find groups in closely related species in prokaryotic strains. PanOCT uses conserved genes neighborhood information to separate recently diverged homologs that standard methodologies fail to find (Fouts et al., 2012). For this, the program unifies various types of methodologies in its flowchart, including protein BLAST (BLASTP) all vs. all, RBH, and BLAST Score Ratio (BSR) to detect orthologous groups (Fouts et al., 2012). There are results of comparison between PanOCT and three commonly orthologous-search tools in commonly used graphs (InParanoid, OrthoMCL, and Sybill) using bacterial strains data available to the public and among them, it turned out that a high relationship between the results obtained, about 86% (Fouts et al., 2012).

The tool makes co-orthologous clusters preferable for this type of analysis. Written in PEARL language, PanOCT is available in Linux OS and it is still in development. It is available in 3.23 version (September 2015 data).

### PhosphOrtholog: a web-based program for mapping ortholog proteins in post-translational modifications into various species

According to homology studies, there is a growing need for tools that facilitate cross-species comparison of PTM data. This is particularly important because functionally modification sites are more likely to be evolutionarily conserved (Chaudhuri et al., 2015). In this context, the web tool PhosphOrtholog was developed. Through an unconventional approach, using proteomic data, PhosphOrtholog works with four major implementations (Chaudhuri et al., 2015) for analyzing data reference maps of orthologs: Presentation Layer, Request Manager Layer, Analysis Layer, and Data Storage Layer.

This application is designed for mapping known and new orthologous PTM sites from experimental data obtained from different species in a large-scale PTM sites. Built on jQuery, Python and R this tool was incorporated and designed in HyperText Markup Language 5 (HTML5) and available exclusively via Web.

### PorthoDom: domain similarity based in orthology detection

In order to minimize the time and computational requirements in comparative analyzes between various sequences of proteins that are available, there is the porthoDom tool. The tool is based on the functional similarity domain of the protein content but their way of comparison is to bring two new measures of similarity between proteins: cosine similarity (COS) measure and a maximal weight matching score. A COS measure is implemented to compute the distance between two domain arrangements of any length (Bitard-Feildel et al., 2015). The cosine measure is a similarity measure often used for high dimensional spaces and the measures show that domain content similarities are able to correctly group proteins into their families (Bitard-Feildel et al., 2015). By using domains instead of amino acid sequences, the reduction of the search space decreases the computational complexity of an all-against-all sequence comparison (Bitard-Feildel et al., 2015).

PorthoDom has a higher performance than proteinOrtho ortholog tool, being 40% faster (Bitard-Feildel et al., 2015). It is a python wrapper that uses protein domain to speed up proteinOrtho. It performs the domain annotation of their protein sequences, or one can use the existing annotation in Pfam format. The clusters are used as orthologs subspace search candidates, that is, sequences of proteins with similar domain arrangement are grouped by species and it is a tool for groups flexibly orthologs through a parameter adjustment according to the user's interest and it makes the process more automated. The implementation of porthoDom is released using Python and C++ languages and is available under the GNU GPL license 3 but it has not been updated since march 2011. PorthoDom is laborious to runs because uses several tools that need to be installed independently such as pfamscan, JAVA, Python, boost C++, BLAST+ and protheinOrtho algorithms.

### PorthoMCL: uses MCL parallel orthology for prediction of orthologous groups in massive genome dataset

PorthoMCL is a orthology predictor that uses MCL for the realm of massive genomes (Tabari and Su, 2017). It is similar to that of OrthoMCL, however, depends on an external database server, that uses a sparse file structure for more efficient data storage (Tabari and Su, 2017). It is increases the number of genes using the “All-Against-All” BLAST and MCL methodologies to scan orthology (Tabari and Su, 2017). The porthoMCL strategy is divided in three main steps: (1) PorthoMCL makes “all-against-all” BLAST searches performing “individual-against-all” BLAST searches in parallel for each genome independently then it identifies the best between the genomes BLAST hits for each two genomes scanning the “individual-against-all” BLAST results; (2) the algorithm finds RBH between two genomes and calculates the normalized score in parallel (second Tabari et al. this is the most computationally intensive step of the algorithm); and (3) PorthoMCL finds within genomes RBH and normalizes the score with the average score of all the paralog pairs that have an orthologs in other genomes (Tabari and Su, 2017). The outputs of these steps are used to construct a sequence similarity graph that is then cut by the MCL program to predict orthologous and paralogous groups (Tabari and Su, 2017).

The program runs on Linux/Unix (OS X) and Windows systems and requires PERL, BLAST, Python, MCL dependences.

### ProteinOrtho: detection of co-orthologs groups in large set of data

The main objectives of the developers of proteinOrtho were to significantly reduce the amount of memory required for orthology analysis of proteins, (being comparatively as good as OrthoMCL and Multi-Paranoid) and to deal easily with a large volume of data. It is implements a BLAST-based approach to determine sets of co-orthologous proteins or nucleic acid sequences that generalizes the reciprocal best alignment heuristic (Lechner et al., 2011). ProteinOrtho is an autonomy and a handling large bacterial datasets using distributed computing techniques when running on multi-core hardware. The performance is comparable with OrthoMCL, due to its low computational request, ease of usage and good efficiency.

ProteinOrtho is one of the most cited in scientific papers and it is available in 5.11 version. It is easily used on Linux via terminal, but needs python, Perl, and BLAST + pre-installed and requires a 64 bit OS.

### ReMark: an automatic method combining a recursive and a markov clustering algorithms

Identifying orthologs automatically is very useful for functional annotation, and studies on comparative and evolutionary genomics (Kim et al., 2011). The program ReMark is a fully automatic tool for clustering orthologs by combining a Recursive and a Markov clustering (MCL) algorithms (Kim et al., 2011). This tool is divided in two main steps: (1) The ReMark detects and recursively ortholog clusters through reciprocal BLAST best hits between multiple genomes running software program (RecursiveClustering.java). (2) Then it employs MCL algorithm to compute the clusters (score matrices generated from the previous step) and refines the clusters by adjusting an inflation factor running software program (MarkovClustering.java) (Kim et al., 2011).

The program was developed in Java scripts, it works in cross-platform, since with the JVM pre-installed on machine.

### SPOCS: a pipeline for predicting and visualizing of orthology and paralogy relationships among genomes

Species Paralogy and Orthology Clique Solver (SPOCS) implements a graph-based orthology prediction method to create a simple tab-delimited table of orthologs and HTML files that provide a visualization of the predicted in orthologs or paralogs relationships to which sequences of genes or proteins (Curtis et al., 2013). SPOCS proceeds in three main stages (Curtis et al., 2013): (1) it executes a series of BLAST runs between every pair of species to identify RBH, allowing subsequent SPOCS runs that include some of the same *n* species to avoid performing BLAST if they already exist; (2) SPOCS uses the BLAST results to generate an orthology or paralogy relationships based on graph merging the pairwise ortholog and in-paralog relationships; and (3) SPOCS identifies cliques in each graph using the branch and bound clique-finding algorithm.

It is a flexible method for quickly and accurately predicting orthologs expression. Another plus point of the tool is that it can be worked via the web, and also locally in Linux or Mac OS X, but dependent on the boost C++ libraries and the previously installed BLAST.

## Results and final considerations

Our critical analysis took into consideration the use of available Orthologs prediction tools gathering information from their presentations and their creators. We highlighted their differences and pointed out the criticisms made about the disadvantages and usability of each one of them.

Hieranoid tool uses hierarchichal approaches to infer orthology using the bit-score method based on the InParanoid algorithm. It computes the orthology graph of a protein through from the global alignment of the sequence with a minimum overlap of 50%. This, though entails a disadvantage as the re-arrangements of orthologs with an extensive domain may be missed. Future plans, announced by the developers of this tool, include the use of domain information in order to fix this failure. The most interesting thing about this pipeline is that the search for similarity can be adjusted using BLAST or the Usearch algorithm.

MorFeus is a software aiming at detecting orthologs when it is difficult to find orthology relationships among evolutionarily distant sequences. It runs on the Web, but the sequence can serve as an input only if it possesses an ID of the RefSeq. The configuration options allow to pick a particular kingdom (Archae, Bacteria, Fungi, Metazoa) or the whole dataset, the default *e*-value is 100 and the output is sent to the email address registered by the user. Unfortunately the local version has not been updated since March 2014 and it requires pre-installed software such as python, biopython, networkx, gnuplot, and BLAST + besides the need of the registration of the user via Web.

OrthAgogue is a tool that was developed in order to predict orthology among large sets of data. It is available in 32 and 64-bit versions and up to the 1.0.2 version (the last update was made in July, 2013). The program relies on the library called Intel Threading Building Blocks (Intel TBB) and on the one called C Minimal Perfect Hashing Library (or library hash CMPH). Thread number settings and threshold overlap are some of its features operating in the agility of the pipeline. It is a tool relatively easy to use because it does not rely on very elaborate prerequisites, however it needs input files in a tabular format, generated by the BLAST algorithm.

OrthoInspector is a tool that offers just one simple and fast algorithm to detect orthology and in-paralogy. It is currently available in its 2.21 version (updated in August, 2015). However, prerequisites that this tool requires turn it cumbersome to operate. For example the entry file in XML format, the BLASTP+ package, the creation of the data set in PostgreSQL or in MySQL, besides the addition of the JAVA package.

OrthoFinder was developed to solve fundamental biases in genome comparisons, improving the accuracy of the inference of orthologous groups. It works as a single command that takes as input a directory of FASTA files (one per specie) and, with the help of statistical algorithms, it generates output files containing genes of orthologous groups of these species. This mechanism is interesting because it seeks to minimize the bias of the length, previously undetected in the genes grouped in orthologs, resulting in significant improvements in the accuracy of the results. It is now at its 1.1.10 version (updated in September 2017) and it depends on the following packages: Python, BLAST+, MCL graph clustering algorithm, MAFFT, and FastTree.

Ortholog-Finder is a tool developed to build ortholog data sets for phylogenetic analysis. Results published by its developers suggest that it can tolerate gene loss after gene duplication and HGT, because most of the phylogenetic trees are accurately reproduced even when these events occur. The algorithm is written in PERL, compatible with Linux/Unix platforms and it needs BLAST, ClustalW, MAFF, and BioPERL to be run. However, this program does not support the maximum likelihood or the Bayes methods.

Orthograph counts on its specific algorithm in order to solve earlier implementations of RBH strategies with graph-based mapping while maintaining the high sensitivity and accuracy of the RBH approach. This tool needs various softwares such as BLAST, SWIPE, MAFF, MySQL in order to be run.

Orthonome was developed to bring more accuracy to orthology methods. Its pipeline counts on the MSOAR method in order to classify groups of homologs. It was proved to have advantages on complete and draft genomes in Drosophilid genomes and it combines multiple pipelines to accuracy and recall of ortholog assignments. It is available on a Web-server basis and this limits the number of inputs. Furthermore, the quality of its assembly and annotations has an impact on its performance, making therefore convenient to take reference genomas as a comparative data set.

OrthoVenn, uses the interactive Venn diagram to generate views of clusters. It is a Web-server based tool searching for orthology between multiple sequences among different species. The user can select up to six species and analyze them against the dataset available in the OrthoVenn Website. This tool conveys gene ontology information with each protein function relating to the respective clusters generated. The pipeline features various methodologies to achieve inference of groups such as BLAST “all-vs.-all,” MCL, and even a predictor of hypothetical proteins, which makes this tool somewhat slow and it implies a limited number of queries for analysis.

PanOCT derives its peculiarities from the fact that it was developed in order to avoid using traditional methods in graph-based detection of orthologs and because it is considered a high-output kind of tool. It uses conserved gene neighborhood (CGN) strategy to improve accuracy in the clusters generated by the algorithm for pan-genomic analysis of prokaryotic species or closely related strains. Therefore, it presents difficulty in detecting groups when organisms are evolutionarily too far apart. It uses PERL packages, BLAST + and it is limited to an analysis of up to 25 genomes providing that the hardware used for analysis has a minimum of 14 GB of RAM available. It closely resembles clustering tools with several interesting execution options such as *e-value* threshold, cut-off identity, creation of files with paralogs groups, BLAST standardization score histogram, window size on either side of match to use CGN, among others. The developers believe that the various options are necessary due to the preference of the orthgroup the co-orthologs with the same genomic context, and additional information need to be reported indicating co-ortholog relationships. It is currently available in the 3.23 version (last updated in July 2016).

Phosphortholog is another tool available exclusively on the Web for mapping types of proteins derived from PTMs. To this end, it uses the UniProt/Swiss-Prot database where information about proteins is collected and its algorithm is score-based on the BLOSUM62 matrix for alignments among sequences. One of the weak points is that the input is in comma separated file format and is restricted to just the proteomas of humans, mice, rats, flies.

ProteinOrtho significantly reduces the amount of memory required for orthology analysis in comparison with existing tools (OrthoMCL and Multi-Paranoid). It finds co-orthologs in large sets of data, containing different species, specifically designed to handle hundreds of species at the same time, containing millions of proteins. However, it is still based on BLAST +, PERL, and Python libraries to run. It is available in its 5.1 last updated in April 2016 version.

PorthoDom is based on the functional similarity domain of the protein and is able to detect it through features two new measures of similarity: cosine similarity (COS) and a maximum weight matching score. It combines Recursive and Markov clustering (MCL) algorithms and it uses the Reciprocal BLAST Best Hits (RBBH) model among multiple genomes running *RecursiveClustering.java* software on the first step. Therefore, it employs the MCL algorithm to calculate the clusters (scoring matrices generated from the previous step) and it refines the adjusting inflation factor by running *MarkovCLustering.java* software.

PorthoMCL is a fast tool that needs low requirements for identifying orthologous and paralogous groups in genomes. It is much faster and more modulable tool as compared to OrthoMCL using the same mathematical fundament to investigate orthology. PorthoMCL can facilitate comparative genomics analysis through a large number of sequenced genomes but it still requires BLAST, PERL, and Python package to be run.

ProteinOrtho is performed with machine distributive techniques to run handling large quantities of data. The different results of the proteinOrtho are gathered and a default output file proteinOrtho is created. Therefore, the tool is a bit elaborate to be performed and requires pre-installed python and HMMER packages, besides the Pfam database.

ReMark is an automatic program to multiple genomes that combines Recursive and Markov Clustering (MCL) algorithms by its pipeline wrote in Java scripts and is not updated since march 2011. It works in cross-platform, since with the JVM pre-installed on machine. BLAST+ algorithms are needed to be runs.

SPOCS arose as an alternative to automate the process of orthologs detection, without the need of multiple steps thanks to its pipeline and it is also considered a tool with low hardware requirements. This tool requires a minimum of 8 GB RAM and Quadcore processing in 64-bit based systems. The software requires a set of protein FASTA files (one per genome species), and an optional additional FASTA file to serve as an outgroup, i.e., a species that should be more distantly related to the species under examination than any of these same species are to each other. BLAST is required to generate the reciprocal best hit results for every pair of species. SPOCS then merges these results identifying orthologs using the graph-based concept of cliques. SPOCS needs boost C++ and BLAST previously installed and it generates text or HTML outputs. The BLAST algorithm is required to generate RBH results for each pair of species. Following that SPOCS combines those results identifying orthologs using graph-based SPOCS produces text or HTLM outputs.

We made a comparison among the different orthologs prediction tools focusing on four objects of their analysis among the main ones in orthology in order to test their performance. Some tools use the same kinds of input data while others require some degree of manipulation of the data being submitted, which would produce a bias in the detection of clusters. In order to avoid that problem, we submitted to the comparison test only those tools that use uniform datasets. Therefore, the criterion for choosing the tools to be submitted to the comparison test consisted in the number of citations the group classification for ortholog prediction and we selected the softwares that use the FASTA format as default input in order to obtain more accurate results, that are described in the Supplementary Table 1. Based on this comparison, we selected proteinOrtho as representative of the tools developed in order to process large sets of data; orthAgogue to represent the higher speed tools; orthoFinder to represent the accuracy tools and ReMark the pipeline tools. In order to be tested by these tools we selected four (complete) proteobacteria genomes deposited in the NCBI databank: *Acidithiobacillus ferrooxidans* ATCC 23270, complete genome (NC_011761), *Desulfitobacterium hafniense* DCB-2 chromosome, complete genome (NC_011830), *Geobacter uraniireducens* Rf4 chromosome, complete genome (NC_009483), and *Herbaspirillum seropedicae* SmR1 chromosome, complete genome (NC_014323).

The complete genomes presented a total of 17,123 proteins, composed of: 3,147 proteins in *A. ferrooxidans*; 4,358 proteins in *G. uraniireducens*; 4,883 proteins in *D. hafniense;* and 4,735 proteins in *H. seropedicae*. (amounting to 17,123 proteins analyzed.) The four tools were compared by number of generated ortholog groups, by number of clusters shared by all species and by singletons (unicast) clusters of each species. Additional information about configuration tools and details about results are available in the Supplementary Table 1. The ReMark tool generated 2,138 orthologous clusters, 21.1% were clusters shared by all species and 12.1% were single copy clusters in a running time of 36.45 min. ProteinOrtho obtained 2,318 orthologous clusters, 17.5% were clusters shared by all species and 11.4% single copies in 10.25 min. OrthAgogue scored 1,926 orthgroups being 18.5% shared by all species and 13.9% by only one in 04.45 min. OrthoFinder gerated 2,230 orthologous clusters, 21.2% shared by all species and 12.4% singletons clusters in 22.00 min of execution.

## Conclusion

The need for developing new tools for the prediction of orthology has been growing in the past decade. By comparing the various available tools we could assess the features and the advantages and flaws of each one of them.

We divided orthology prediction tools in four main categories: (1) the ones designed to achieve more accuracy; (2) those aimed at higher speed orthologs detection; (3) the ones more fit for handling large data sets; and (4) the pipelines (integration methods) in orthology analyses. Meanwhile, some problems still persist and should be solved by more recent methodologies. For instance, most tools still use the BLAST algorithm, which demands more processing memory thus restricting the number of sequences that can be analyzed even with new methodologies. In order to represent the four groups of tools mentioned above we selected ReMark, ProteinOrtho, OrthAgogue, and OrthoFinder and submitted to them four bacteria genomes. The result was that ReMark, representing pipeline-based tools, detected a number of clusters shared by all the species comparable to OrthoFinder, the accuracy tool that obtained the best result but taking a lot of time to perform its task. ProteinOrtho, representing the tools developed to tackle large number of data, detected the highest number of clusters as compared to the others. And, finally, OrthAgogue detected the largest number of clusters taking the shortest running time.

We hope that our short review might be of use for those working in the field of orthologs analysis and help in the improvement of existing orthologs detection tools and in the development of future ones.

## Author contributions

BN the principal author, contributed in the preparation and writing, and all feedback to the work doing academic research. RR co-author reviewer and supervisor of paper, methodology, revising it critically and participated in the article writing. JM co-author reviewer in the English language article, analysis critic and paper workflow.

### Conflict of interest statement

The authors declare that the research was conducted in the absence of any commercial or financial relationships that could be construed as a potential conflict of interest.
