# Supplementary material for: New Tools in Orthology Analysis: A Brief Review of Promising Perspectives
Source: Front Genet. 2017 Oct 31;8:165. doi: 10.3389/fgene.2017.00165 (PMC5674930; doi:10.3389/fgene.2017.00165)
Supplement: Supplementary file 1 [file Table1.PDF]

Supplementary informations

New Tools in Orthology Analysis: A Brief Review of Promising Perspectives

Bruno Thiago de Lima Nichio[1], Jeroniza Nunes Marchaukoski[1] and Roberto Tadeu Raittz[1]

Affiliations:

[1]Department of Bioinformatics, Professional and Technical Education Sector from the University Federal of Paraná, Curitiba, PR, Brazil

Correspondence should be addressed to Roberto Tadeu Raittz (raittz@ufpr.br)

Supplementary Graphics

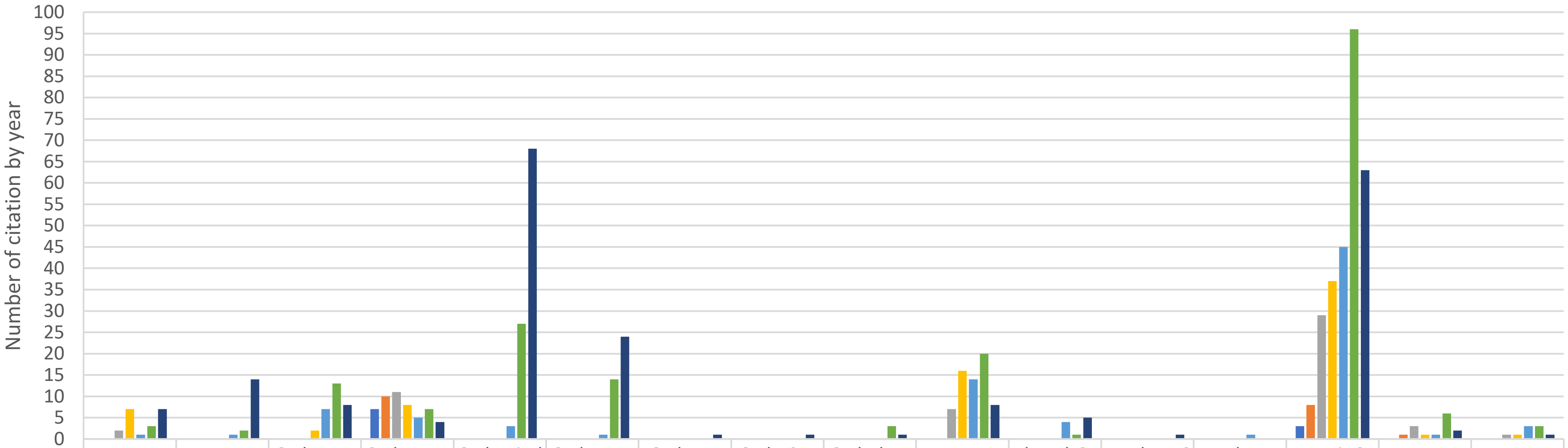

	Hieranoid	morFeus	OrthAgogue	OrthoInspector	OrthoFinder	OrthoVenn	Orthonome	OrthoScape	Ortholog-Finder	PanOCT	PhosphOrtholog	PorthoMCL	PorthoDom	ProteinOrtho	ReMArk	SPOCS
2011				7										3		
2012				10										8	1	
2013	2			11						7				29	3	1
2014	7		2	8						16				37	1	1
2015	1	1	7	5	3	1				14	4		1	45	1	3
2016	3	2	13	7	27	14			3	20	1			96	6	3
2017	7	14	8	4	68	24	1	1	1	8	5	1		63	2	1

Ortholog tools

Figure 1 - Growth of Number of the citations by each ortholog tool from 2011 to 2017. A brief relationship between the number of citations per year for each tool. It is observed that some of the tools have citations more than others in other, such as, ProteinOrtho, OrthoInspector, OrthoFinder and PanOCT revealing a good acceptance of tools in other works across the years totalizing more than 640 papers filtered and analyzed.

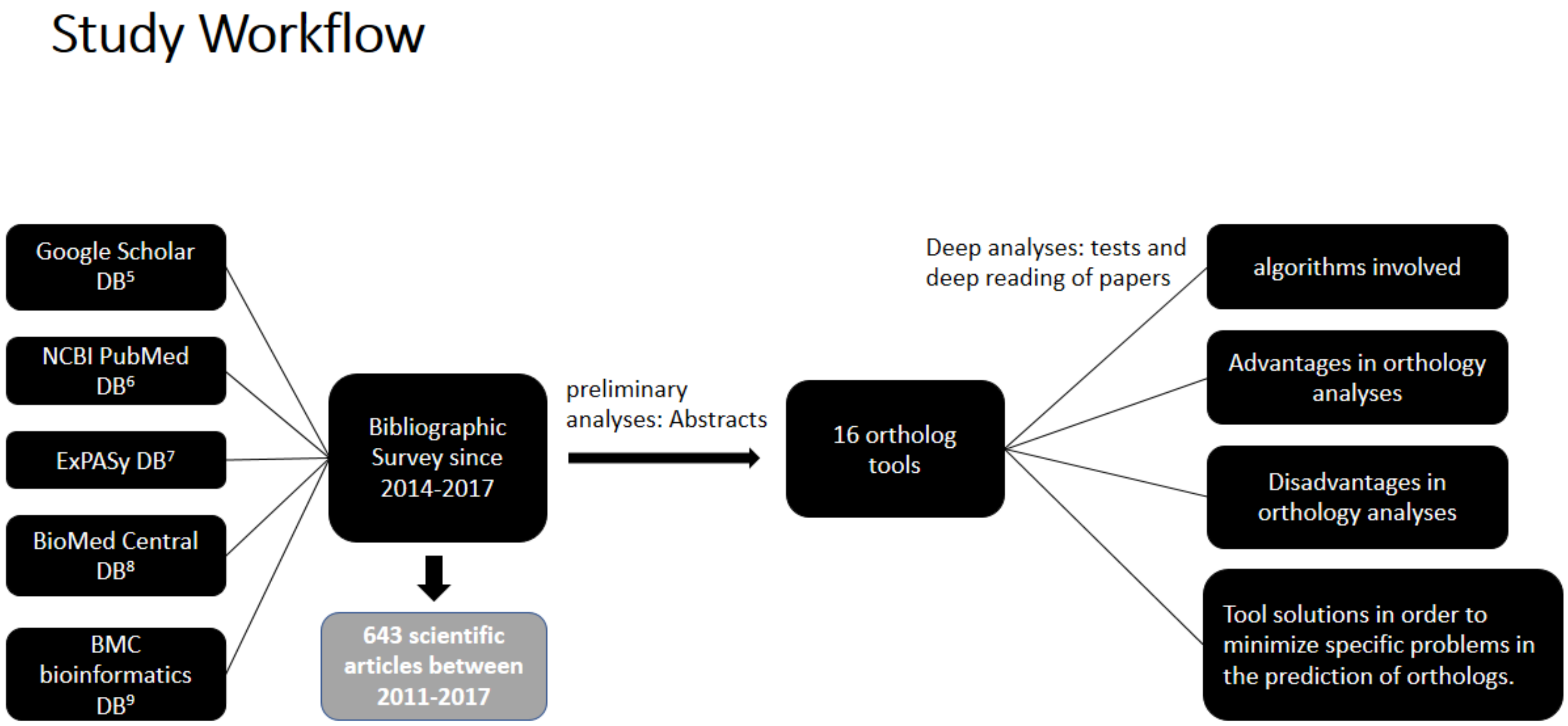

Figure 2 – Filtering workflow to select the ortholog tools: Bibliographic survey made between 2014-2017 selecting recent tools since 2011 until present day. 643 papers are revised in a **preliminary analyses** to appoint the tools usage, algortithms envolved relationed with orthology prediction. The **deep analyses** appoints to the main characteristics in each tool, advantages or desadvantages and and solutions in order to minimize some specific problem in orthology studies.

Supplementary Tables

TABLE 1 – Comparison tool between different main features in orthology analysis*

Main feature	Representing tool	Usage	Total clusters	Orthologous clusters (Orthgroups)**	Clusters shared by all species	Singletons Clusters (Single copy)	Running Time (min)
**Pipeline**	ReMark[1]	Linux/Windows command line	3230	2138 (66,2%)	681 (21.1%)	411 (12.7%)	37.45
**Applicable in large data sets**	ProteinOrtho[2]	Linux command Line	3261	2318 (71,1%)	573 (17.5%)	370 (11.4%)	10.25
**Higher speed**	OrthAgogue[3]	Linux command line	2847	1926 (67,6%)	531 (18.5%)	390 (13.9%)	04.45
**Accuracy**	OrthoFinder[4]	Linux command line	3358	2230 (66,4%)	711 (21.2%)	417 (12.4%)	22.00

* The results are obtained in a machine with i5-650 3.20GHz dual core and 16Gb RAM in a Linux OS (Ubuntu 16.04 LTS) x64 bits. The programs are performed with default configurations and applied in bacteria genomes deposited on NCBI databank *Acidithiobacillus ferrooxidans* ATCC 23270, complete genome (NC_011761), *Desulfitobacterium hafniense* DCB-2 chromosome, complete genome (NC_011830), *Geobacter uraniireducens* Rf4 chromosome, complete genome (NC_009483) and *Herbaspirillum seropedicae* SmR1 chromosome, complete genome (NC_014323).

**Clusters gerated by at least two species

References:

1 Kim, K., Kim, W., & Kim, S. (2011). ReMark: An automatic program for clustering orthologs flexibly combining a Recursive and a Markov clustering algorithms. *Bioinformatics*, *27*(12), 1731–1733. http://doi.org/10.1093/bioinformatics/btr259

2 Lechner, M., Findeiß, S., Steiner, L., Marz, M., Stadler, P. F., & Prohaska, S. J. (2011). Proteinortho: Detection of (Co-)orthologs in large-scale analysis. *BMC Bioinformatics*, *12*(1), 124. http://doi.org/10.1186/1471-2105-12-124

3 Ekseth, O. K., Kuiper, M., & Mironov, V. (2014). OrthAgogue: an agile tool for the rapid prediction of orthology relations. *Bioinformatics*, *30*(5), 734–736. http://doi.org/10.1093/bioinformatics/btt582

4 Emms, D. M., & Kelly, S. (2015). OrthoFinder: solving fundamental biases in whole genome comparisons dramatically improves orthogroup inference accuracy. *Genome Biology*, *16*(1), 157. http://doi.org/10.1186/s13059-015-0721-2

5 Google Schoolar Database articles session [internet]. Google Databank (1998). [cited 29 september 2017] <https://scholar.google.com/>.

6 U.S. National Library of Medicine Database [internet]. Bethesda (MD): National Center for Biotechnology Information, U.S. National Library of Medicine (1988) [cited 29 september 2017] <https://www.ncbi.nlm.nih.gov/pubmed/>

7 Expert Protein Analysis System Database. ExPASy: SIB bioinformatics resource portal (1993) [cited 29 september 2017] <https://www.expasy.org/>

8 BioMed Central (BMC) database. Springer Nature (1999) [cited 29 september 2017] <https://www.biomedcentral.com/>

9 BioMed Central (BMC) database session bioinformatics. Springer Nature (1999) [cited 29 september 2017] <https://bmcbioinformatics.biomedcentral.com>
